# Artificial intelligence in cancer care: Opportunities and challenges from a nursing perspective

**DOI:** 10.1016/j.apjon.2026.100935

**Published:** 2026-03-06

**Authors:** Tuzhen Xu

**Affiliations:** College of Nursing, Prairie View A&M University, TX 77030, USA

Artificial intelligence (AI) is rapidly reshaping oncology, offering new opportunities to enhance diagnostics, treatment decision-making, care planning, and patient outcomes across the cancer care continuum.^1^ While much of the current discourse has focused on physician-led applications of AI, oncology nurses occupy a uniquely influential position at the intersection of advanced technology and direct, patient-centered care. Although nursing perspectives remain underrepresented in the AI literature, emerging work suggests that AI-enabled tools may meaningfully support symptom management, strengthen communication with patients and their families, inform nursing care planning, and ultimately improve patient outcomes. These applications have particular relevance for nursing practice, as they directly influence ongoing assessment, patient communication, and care coordination, which are central to effective and compassionate oncology care.

## AI and oncology nursing practice

In nursing practice, the value of AI lies less in replacing clinical judgment and more in its capacity to extend nursing surveillance, optimize workflows, and support more timely and informed clinical decision-making. When integrated into electronic health records, AI-driven tools can help oncology nurses identify early signs of treatment-related toxicity or clinical deterioration, enabling more proactive, coordinated intervention.^2^ The capability is particularly salient in cancer care, where patients may be unable to verbalize symptoms due to disease burden, cognitive changes, or serious illness, leaving critical aspects of suffering underrecognized in traditional care models. Emerging AI models that extract clinically meaningful signals from structured and unstructured clinical records offer a way to surface non-visible symptoms, including pain, dyspnea, fatigue, anxiety, delirium, and spiritual distress.^3^ By reducing reliance on time-intensive questionnaires and episodic assessments, these tools have the potential to alleviate nursing workload while enhancing the continuity and depth of symptom evaluation. Referral systems powered by AI have resulted in more timely referrals among cancer patients, with electronic health record-embedded triggers significantly increasing early palliative treatment or consultation rates. It further demonstrates that these AI-driven insights help nurses proactively develop nursing care plans and improve overall nursing quality. Importantly, such applications do not replace nursing assessment but amplify nurses’ ability to recognize complex patterns of distress and respond with targeted, compassionate care.

Beyond symptom surveillance, AI applications that support communication and clinical decision-making are rapidly evolving, with important implications for nursing practice. For instance, analytic tools that review clinical documentation to prompt goals of care discussions or advanced care planning may help address longstanding gaps in timely, values-based conversations.^4^ This function may be particularly meaningful in cultural contexts where discussion of serious illness and end-of-life care is stigmatized or avoided, such as in parts of the Asia–Pacific region. In these settings, AI-informed prompts guided by nursing judgment may serve as catalysts for earlier, more culturally sensitive engagement rather than substitutes for human dialogue.

At the same time, AI offers emerging opportunities to evaluate and improve the quality of nursing care itself, shifting the focus from task completion to meaningful outcomes through workflow optimization and innovations in nursing training and education. AI-enhanced simulation training systems, including virtual and robotic-assisted training environments, have shown promise in strengthening decision-making and situational awareness among nurses.^5^ Rather than replacing experiential learning, these tools may complement traditional education by providing scalable, adaptive, and data-informed training opportunities that better prepare nurses for the complexity of contemporary oncology care. As AI becomes more embedded in clinical practice, its role in shaping how nurses are trained, evaluated, and supported will be central to sustaining high-quality, patient-centered care. However, the benefits of these advanced technologies will only be realized if they are thoughtfully integrated into nursing workflows and governed by nurses’ clinical judgment, ensuring that AI supports rather than supplants professional nursing practice.

## Patient and family implications

For patients with cancer who receive most of their care outside of hospital settings, AI-enabled technologies offer new possibilities to address the longstanding gap in continuity of care. Through remote monitoring and telehealth, AI can extend nursing oversight beyond clinical settings and into patients' homes, which may be particularly impactful for individuals living in rural or underserved areas. AI-driven tools, including wearable devices and sensor-based systems, can continuously track physiologic indicators and symptom trajectories, alerting nurses to patients’ early signs of clinical deterioration. Meanwhile, chatbots or other AI-mediated communication platforms may enable patients and family caregivers to engage in timely, real–time interactions with the care team, supporting symptom reporting, education, and reassurance between scheduled visits. Rather than focusing solely on early detection, these technologies may reshape how patients and families experience cancer care by offering greater continuity, responsiveness, and perceived support outside of traditional clinical encounters.

Beyond gains in clinical efficiency, the integration of AI into oncology care also brings meaningful psychosocial implications for patients and their families navigating the uncertainty and emotional burden of serious illness. For patients and caregivers managing complex symptoms at home, particularly in rural or resource-limited settings that have limited access to in-person support, AI-enabled monitoring can reduce uncertainty about changes in condition and mitigate the sense of isolation often associated with home-based cancer care. When thoughtfully implemented, these tools may strengthen patients' and caregivers’ sense of connection to the health care team, alleviate psychological distress, and support emotional reassurance through timely nursing engagement. However, continuous monitoring may also introduce unintended consequences, including heightened anxiety, increased caregiver burden with new responsibilities, or unrealistic expectations of constant clinical availability. Patients and families may also differ in their comfort with digital surveillance, data sharing, and algorithm-informed decision-making.

From a nursing perspective, AI-enabled care must be implemented in ways that preserve autonomy, respect cultural values, and reinforce the therapeutic nurse–patient relationship. Nurses play a critical role in contextualizing algorithmic outputs, interpreting alerts within patients’ lived experience, and addressing the emotional responses that may arise from continuous monitoring. Recent studies have extended AI-enabled documentation to language translation to help patients and families with limited English proficiency. While such tools cannot substitute for professional interpretation at this stage, they may enhance health care access, understanding, and equity when integrated under nursing oversight. Moving forward, the success of AI in oncology care depends not only on technical performance but on its ability to support holistic, person and family-centered care grounded in nursing values.

## Equity and ethics considerations

As AI becomes increasingly embedded in cancer care, its ethical and equity implications demand careful scrutiny, particularly from a nursing perspective grounded in patient advocacy and relational care. Most AI systems in oncology were developed using retrospective, single-center datasets, at a developmental stage, with relatively few studies undergoing real-world or prospective validation.^6^ Moreover, longstanding inequities related to race, socioeconomic status, language proficiency, and access to care are well documented within oncology datasets, raising concerns that biases may be inherited and amplified by AI-driven tools. While some may argue that AI may reduce subjective bias by standardizing decision-making, AI-informed recommendations, such as risk stratification, symptom prioritization, or care escalation, may disadvantage populations already experiencing inequitable care. From a nursing perspective, ethical AI integration requires representative data, ongoing performance monitoring across diverse patient groups, and human-in-the-loop oversight for high-stakes clinical decisions. Nurses are uniquely positioned to recognize when algorithmic recommendations conflict with patients’ lived experiences, values, or goals of care, and to intervene accordingly.

In addition to bias, AI raises broader ethical questions related to autonomy, transparency, and accountability. Overreliance on algorithmic guidance may unintentionally diminish clinicians’ critical judgment or constrain meaningful shared decision-making with patients and families. Medico-legal uncertainties surrounding AI-generated recommendations further complicate implementation, particularly when predictive outputs influence treatment trajectories. Clear delineation of responsibility, transparent communication with patients, and explicit framing of AI as a decision-support rather than decision-making tool are essential to preserving ethical standards in oncology nursing practice. Meanwhile, routine exposure to frequent alerts may result in alert fatigue, requiring thoughtful system design that supports rather than distracts from clinical vigilance.

## Future direction

Looking forward, future directions must extend beyond technical refinement to include meaningful engagement with stakeholders across the cancer care continuum. Nurses will require targeted education and institutional support to build digital competencies and interpret AI output appropriately in clinical contexts, while nursing scholars need to deepen their understanding of AI's mechanisms and applications, ideally in collaboration with developers, with an emphasis on nursing-led research. Future research should more deliberately examine the perspectives of nurses, patients, family caregivers, and administrators to assess whether AI tools truly enhance care quality, workflow efficiency, and therapeutic relationships, or whether they introduce new burdens or inequities. Interdisciplinary collaboration among AI developers, clinicians, data scientists, and policymakers will be essential to embedding AI within holistic, person-centered models of care. The successful integration of AI in cancer care also depends on addressing implementation challenges, including variability in digital literacy, interoperability limitations, and unequal resources across care settings. More rigorous research needs to be conducted to evaluate the effectiveness, feasibility, and equity of AI applications in these diverse clinical settings. At a systems level, policies and governance structures must also promote equitable AI deployment, accounting for regional disparities in infrastructure and digital capacity.

## Ethics statement

Not required.

## Data availability statement

Data availability does not apply to this article as no new data were created or analyzed in this study.

## Declaration of generative AI and AI-assisted technologies in the writing process

No AI tools/services were used during the preparation of this work.

## Funding

This study received no external funding.
